# Cellular and humoral mechanisms of osteoclast formation in Ewing's sarcoma

**DOI:** 10.1038/sj.bjc.6603774

**Published:** 2007-05-29

**Authors:** Y S Lau, I E Adamopoulos, A Sabokbar, H Giele, C L M H Gibbons, N A Athanasou

**Affiliations:** 1 Department of Pathology, Nuffield Department of Orthopaedic Surgery, Nuffield Orthopaedic Centre, University of Oxford, Oxford OX3 7LD, UK; 2 Nuffield Department of Surgery, Nuffield Orthopaedic Centre, University of Oxford, Oxford OX3 7LD, UK

**Keywords:** Ewing's sarcoma, macrophage, osteoclast, bone resorption

## Abstract

Cellular mechanisms that account for tumour osteolysis associated with Ewing's sarcoma are uncertain. Osteoclasts are marrow-derived multinucleated cells (MNCs) that effect tumour osteolysis. Osteoclasts are known to form from macrophages by both receptor activator for nuclear factor-*κ*B (RANK) ligand (RANKL)-dependent and -independent mechanisms. In this study, our aim has been to determine whether tumour-associated macrophages (TAMs) isolated from Ewing's sarcoma are capable of differentiating into osteoclasts and to characterise the cellular and humoral mechanisms whereby this occurs. Tumour-associated macrophages were isolated from two Ewing's sarcomas and cultured on both coverslips and dentine slices for up to 21 days with soluble RANKL and macrophage colony stimulating factor (M-CSF). Osteoclast formation from TAMs (CD14^+^) was evidenced by the formation of tartrate-resistant acid phosphatase (TRAP) and vitronectin receptor (VNR)-positive MNCs, which were capable of carrying out lacunar resorption. This osteoclast formation was inhibited by the addition of bisphosphonates. Both Ewing's sarcoma-derived fibroblasts and some bone stromal cells expressed RANKL and supported osteoclast formation by a contact-dependent mechanism. We also found that osteoclast differentiation occurred when Ewing's TAMs were cultured with tumour necrosis factor (TNF)-*α* in the presence of M-CSF and that TC71 Ewing's sarcoma cells stimulated osteoclast formation through the release of a soluble factor, the action of which was abolished by an antibody to TNF-*α*. These results indicate that TAMs in Ewing's sarcoma are capable of osteoclast differentiation by both RANKL-dependent and TNF-*α-*dependent mechanisms and that Ewing's sarcoma cells produce osteoclastogenic factor(s). Our findings suggest that anti-resorptive and anti-osteoclastogenic therapies may be useful in inhibiting the osteolysis of Ewing's sarcoma.

Rapid tumour growth and extensive bone destruction are characteristic features of Ewing's sarcoma of bone and can result in bone pain and pathological fracture (Mirra, 1989; Dorfman and Czerniak, 1998). Although the tumour osteolysis associated with the growth of primary and metastatic tumours in bone is thought to be mediated by osteoclasts rather than tumour cells (Quinn *et al*, 1994; Clohisy *et al*, 1996, 2000; Clohisy and Ramnaraine, 1998), the precise cellular and humoral mechanisms that contribute to the extensive osteolysis of rapidly growing malignancies of bone, such as Ewing's sarcoma, are unknown.

Osteoclasts are specialised, multinucleated, bone-resorbing cells that form by fusion of circulating mononuclear precursor cells of haematopoietic origin (Athanasou, 1996). *In vitro* studies have defined the ontogeny of the osteoclast and characterised the essential cellular and humoral factors, which are required for osteoclast differentiation. In both mouse and man, mononuclear osteoclast precursors circulate in the monocyte fraction and express a monocyte/macrophage rather than a mature osteoclast phenotype (Udagawa *et al*, 1990; Fujikawa *et al*, 1996). These circulating precursors express RANK and their differentiation into osteoclasts requires the presence of macrophage-colony stimulating factor (M-CSF) and involves a receptor–ligand interaction with osteoblasts/bone stromal cells that express the membrane-bound ligand for receptor activator for nuclear factor-*κ*B (RANK), ligand (RANKL) (Tanaka *et al*, 1993; Yasuda *et al*, 1998). Receptor activator for nuclear factor-*κ*B ligand-independent mechanisms of osteoclast formation also exist in which cytokines, such as tumour necrosis factor (TNF)-*α*, a known product of Ewing's sarcoma tumour cells (Rube *et al*, 2003), can substitute for RANKL and induce osteoclast formation from circulating precursors (Kudo *et al*, 2002).

It has been shown that TAMs isolated from carcinomas and sarcomas, when cocultured with bone-derived stromal cells and M-CSF, are able to differentiate into multinucleated osteoclasts capable of extensive lacunar resorption (Quinn *et al*, 1998; Yang *et al*, 2002). This process is RANKL-dependent and is of interest with regard to tumour osteolysis associated with Ewing's sarcoma as TAMs form a significant inflammatory component of rapidly growing malignant tumours (Bugelski *et al*, 1987; Walter *et al*, 1991; Mantovani *et al*, 1992; Van Ravenswaay *et al*, 1992).

The pathogenic mechanisms that account for the extensive tumour osteolysis accompanying the enlargement of rapidly growing sarcomas, such as Ewing's sarcoma, are unknown. In this study, our aim has been to analyse the cellular and molecular mechanisms which contribute to the formation of osteoclasts in Ewing's sarcoma. In particular, we have sought to determine whether TAMs in Ewing's sarcoma differentiate into osteoclasts by a RANKL-dependent or -independent mechanism. We have also examined whether Ewing's sarcoma tumour cells, tumour fibroblasts and bone stromal cells can influence this process.

## MATERIALS AND METHODS

This study was approved by the Oxford Clinical Research Ethics Committee. All cell incubations were performed in *α-*minimum essential medium (MEM) with 10% heat-inactivated fetal bovine serum (FBS), 100 U ml^−1^ penicillin and 10 *μ*g ml^−1^ streptomycin (Gibco, Paisley, UK) (MEM/FBS). All experiments were incubated at 37°C in a humidified atmosphere of 5% CO_2_ and 95% air, and carried out in triplicate, with culture medium and factors replenished every 3–4 days.

M-CSF, TNF-*α*, IL-1*α*, osteoprotegerin (OPG) and anti-human TNF-*α* antibody were obtained from R&D Systems Europe (Abingdon, UK), soluble RANKL was obtained from Peprotech (London, UK), zoledronate was obtained from Novartis (Basel, Switzerland). All experiments were carried out with factors at the following concentrations unless otherwise specified: M-CSF (25 ng ml^−1^), RANKL (30 ng ml^−1^), TNF-*α* (20 ng ml^−1^), IL-1 *α* (10 ng ml^−1^), zoledronate (10^−8^ M), OPG (500 ng ml^−1^), anti-human TNF-*α* antibody (10 *μ*g ml^−1^).

The human Ewing's tumour cell line TC71 was kindly provided by Professor Boshoff (University College London, UK). Ewing's sarcoma tumour tissue was obtained from two cases; a 2 cm^3^ biopsy of the soft tissue component of a tumour of the ilium in a 14-year-old female and a 1 cm^3^ biopsy of the soft tissue component of a tumour of the tibia in a 22-year-old female. Frozen and paraffin sections of each tumour specimen were cut and processed routinely for diagnostic histopathology. Immunohistochemistry with monoclonal antibodies TUK4 and KP1 (Dakopatts, Glostrup, Denmark) was carried out to determine respectively the expression of CD14 and CD68 (monocyte/macrophage-associated antigens), to identify the presence of TAMs in these tumours. The expression of MIC-2 (CD99) was used to identify Ewing's sarcoma cells using monoclonal antibody 12E7 (Dakopatts).

Bone stromal cells were obtained from explants of cancellous bone obtained from hip arthroplasties carried out for osteoarthritis in five patients (three women and two men; age range 58–71 years).

### Isolation and culture of tumour-associated macrophages from Ewing's sarcomas

Tumour-associated macrophages were isolated as described previously (Quinn *et al*, 1998). Fresh tumour tissue was digested with Type 1 collagenase (Sigma-Aldrich, Dorset, UK) for 1 h. The digested tissue suspension was passed through a 70 *μ*m pore-size cell strainer (Falcon®, Becton Dickinson, Oxford, UK). The filtrate was centrifuged at 380 × *g* for 10 min and the cells were counted in a haemocytometer after lysis of red blood cells with 5% (v/v) acetic acid. A total of 1 × 10^5^ cells well^−1^ were added to ivory dentine slices and glass coverslips in a 96-well tissue culture plate. After 2 h incubation, the dentine slices and glass coverslips were washed in MEM/FBS and transferred into 24-well tissue culture plates. All cultures were maintained for 24 h and up to 21 days in the presence of M-CSF and RANKL±zoledronate, or M-CSF, TNF-*α* and IL-1*α*±zoledronate. Negative control cultures were maintained in MEM/FBS with M-CSF.

### Isolation of human peripheral blood mononuclear cells

Human monocytes were obtained by density gradient centrifugation of buffy coat cell preparation (National Blood Transfusion Service, Bristol, UK). The buffy coat preparation was mixed with an equal volume of MEM and purified over Histopaque (Sigma-Aldrich). After centrifugation at 600 × *g* for 25 min, the cell layer above the Histopaque was collected, suspended in MEM and centrifuged at 380 × *g* for 10 min. The cell pellet was resuspended in MEM and centrifuged again. MEM/FBS 5 ml was then added to the cell pellet and the number of cells counted in a haematocytometer following lysis of red blood cells with 5% (v/v) acetic acid. A total of 5 × 10^5^ cells per well were plated onto dentine slices and glass coverslips in 96-well tissue culture plates with MEM/FBS. After 2 h incubation, the dentine slices and glass coverslips were washed in MEM/FBS and transferred into 24-well tissue culture plates containing MEM/FBS and M-CSF. Positive controls were set up in the presence of M-CSF and RANKL.

### Cytochemical and functional assessment of osteoclast formation

Following incubation for 24 h and 14 days, cultures on glass coverslips were fixed and stained cytochemically for the osteoclast-associated enzyme tartrate-resistant acid phosphatase (TRAP) (Minkin, 1982), and immunocytochemically with the monoclonal antibody 23C6 (Serotec, Kidlington, Oxon, UK) for the presence of vitronectin receptor (VNR), an osteoclast-specific antigen (Horton *et al*, 1985) and with MIC-2 for the presence of the Ewing's sarcoma-associated antigen, CD99, using an indirect immunoperoxidase technique (Gatter *et al*, 1984). In addition, these cultures were immunocytochemically stained with the monoclonal antibody TUK4 for the presence of CD14. Functional evidence of TAM-osteoclast formation was determined by a lacunar resorption assay system using cell cultures on dentine slices (Fujikawa *et al*, 1996). After 21 days incubation, the cells were removed from the dentine slices by treatment with 0.1 M ammonium hydroxide. The dentine slices were washed, sonicated and stained with 0.5% (v/v) toluidine blue to reveal areas of lacunar resorption and examined by light microscopy.

### Isolation of Ewing's sarcoma-derived fibroblasts and bone stromal cells and co-culture with PBMCs

After enzyme digestion of the tumour, the cell pellet obtained was resuspended in MEM/FBS and placed in a 25 cm^2^ tissue culture flask; the culture medium was changed after 24-h incubation and then at 5–7 day intervals. These cultures, containing spindle-shaped fibroblast-like cells, were passaged at least three times before they were used for co-culture experiments and RNA extraction as described previously (Lau *et al*, 2006).

Bone stromal cells were obtained from explants of cancellous bone as described previously (Gundle and Beresford, 1995). Briefly, the bone pieces were placed in MEM/FBS and cultured until confluent in a manner similar to that described above for Ewing's sarcoma-derived fibroblasts. The cells were passaged at least three times before they were used for co-culture experiments and RNA extraction.

To determine whether Ewing's sarcoma-derived fibroblasts or bone stromal cells could induce osteoclast formation and to examine if this occurred through the release of a soluble factor or required cell–cell contact, human peripheral blood mononuclear cells (PBMCs) were co-cultured with Ewing's sarcoma-derived fibroblasts (or bone stromal cells) for 21 days in the presence of M-CSF±OPG (Lau *et al*, 2006). Parallel cultures were set up with PBMCs and Ewing's sarcoma-derived fibroblasts (or bone stromal cells) separated by a 0.4 *μ*m Transwell® polycarbonate membrane (Nunc, Invitrogen, Paisley, Scotland).

### Messenger RNA expression of RANKL, OPG and TRAIL

Messenger RNA (mRNA) expression of RANKL, OPG and tumour necrosis factor related apoptosis inducing ligand (TRAIL) was determined in TC71 cells, Ewing's sarcoma-derived fibroblasts and isolated cancellous bone stromal cells. Total RNA extraction was carried out using the RNeasy® mini kit (Qiagen, Hombrechtikon, Switzerland), according to the manufacturer's instructions. Single-strand complementary DNA (cDNA) was synthesised from 2.0 *μ*g of total RNA using the SuperScript® First-Strand Synthesis System for reverse transcriptase–polymerase chain reaction (Invitrogen, Paisley, UK). Complementary DNA was amplified by PCR using the primers shown in Table 1. Polymerase chain reaction products were fractionated on 1% agarose gels, and gel pictures were scanned with AlphaImager 2200 (Alpha Innotech Corporation, San Leandro, CA, USA).

### TC71 Ewing's sarcoma cells: effect on osteoclast formation

TC71 cells were cultured in 25 cm^2^ culture flasks until confluent; conditioned medium (CM) was then extracted after further incubation for 48 h. A dose–response curve (0–20%) established that 10% CM provided the optimal concentration for osteoclast formation (Figure 4A). Subsequent experiments with human PBMCs were set up as follows:

1. M-CSF (negative control)
2. M-CSF, RANKL (positive control)
3. M-CSF, 10% CM
4. M-CSF, 10% CM, ±OPG
5. M-CSF, 10% CM, ±anti-human TNF-*α* antibody

To ascertain if Ewing's sarcoma cells are able to resorb directly bone, parallel 21-day cultures were set up with TC71 cells seeded onto glass coverslips and dentine slices incubated with M-CSF alone, M-CSF and RANKL, or with no added factors.

### Statistical analysis

The amount of lacunar resorption in cell cultures on dentine slices was measured (non-blinded) using image analysis software (Adobe Photoshop, www.adobe.com, USA) as described previously (Quinn *et al*, 1998) and expressed as the mean percentage of surface area resorbed±s.e.m. All resorption data were normalised and expressed relative to the response obtained in PBMC cultures incubated with M-CSF and RANKL (positive control). Statistical significance was determined using the unpaired *t*-test and *P*-values <0.05 were considered significant.

## RESULTS

### Characterisation of TAMs isolated from Ewing's sarcoma

24-h cultures of Ewing's sarcoma mononuclear cells on glass coverslips in the presence or absence of RANKL and M-CSF did not express the osteoclast markers TRAP and VNR but strongly expressed CD14, a macrophage antigen which is known not to be present on osteoclasts (Athanasou and Quinn, 1990; Athanasou, 1996). There was no expression of the Ewing's sarcoma-associated antigen, CD99. In 24-h cultures of isolated cells on dentine slices, no evidence of lacunar resorption was noted. The mononuclear cells isolated from Ewing's sarcomas thus expressed only the phenotypic characteristics of TAMs and not osteoclasts.

### Ewing's sarcoma TAM-osteoclast differentiation

**Figure 1:**
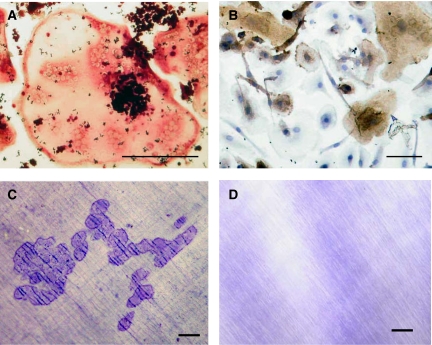
(**A**) TRAP^+^ and (**B**) VNR^+^ MNCs formed after 14 days when Ewing's sarcoma-derived TAMs were cultured in the presence of RANKL and M-CSF. (**C**) Extensive lacunar resorption on a dentine slice after TAMs were cultured for 21 days in the presence of RANKL and M-CSF (Toluidine blue staining). (**D**) No resorption was seen on dentine in 21-day TAM cultures when RANKL was omitted (toluidine blue staining). Zoledronate-treated cultures showed a similar appearance. Bars=50 *μ*m.

Multinucleated cells (MNCs) expressing the osteoclast-associated markers, TRAP and VNR, were found to be present in all 14-day TAM cultures incubated with RANKL and M-CSF (Figure 1A and B). Scattered mononuclear cells positive for CD14 were also noted in these cultures, indicating that not all TAMs showed evidence of osteoclast differentiation. The TRAP+/VNR+ mononuclear cells were also noted in these cultures; mononuclear cells that exhibit the cytochemical and functional characteristics of osteoclasts have previously been noted in mouse and human macrophage cultures (Sterling *et al*, 1998; Quinn *et al*, 2002; Adamopoulos *et al*, 2006). There was no expression of CD99. No TRAP^+^ or VNR^+^ MNCs were seen when RANKL was omitted or when zoledronate was added to M-CSF/RANKL-treated cultures. The TRAP^+^ and VNR^+^ MNCs were also noted in cultures of TAMs incubated in the presence of TNF-*α* and M-CSF.

Cultures of Ewing's sarcoma TAMs incubated on dentine slices for 21 days in the presence of M-CSF/RANKL or TNF-*α*/IL-1*α* showed a functional evidence of osteoclast differentiation with the formation of several areas of lacunar resorption on all dentine slices. Resorption was evident as discrete areas of osteolysis composed of single resorption pits (TNF-*α*/IL-1*α*-treated) or multiple compound areas of lacunar excavation (M-CSF/RANKL-treated) (Figure 1C) on the surface of each dentine slice. Lacunar resorption was not seen when M-CSF or soluble RANKL was omitted from the cultures (Figure 1D). When zoledronate was added to M-CSF/RANKL or TNF-*α*/IL-1*α*-treated cultures, no resorption was seen on dentine slices.

### Effect of Ewing's sarcoma-derived fibroblasts and bone stromal cells on osteoclast formation

**Figure 2:**
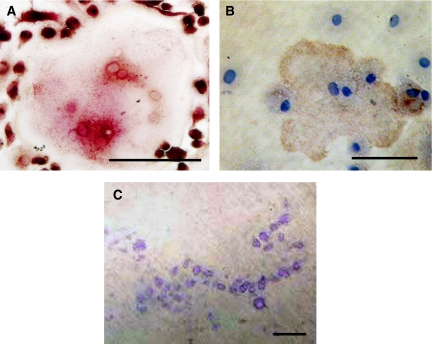
(**A**) TRAP^+^ and (**B**) VNR^+^ MNCs in a 14-day coculture of human PBMCs with Ewing's sarcoma-derived fibroblasts in the presence of M-CSF. (**C**) Lacunar resorption pits formed on a dentine slice in a 21-day coculture of human PBMCs with Ewing's sarcoma-derived fibroblasts in similar conditions. Bars=50 *μ*m.

Fibroblasts isolated from Ewing's sarcomas and bone stromal cells consisted almost entirely of spindle-shaped mononuclear cells, which did not stain immunohistochemically for CD99, CD14 or CD68 or VNR and TRAP. When CD14^+^ monocytes were co-cultured with either Ewing's sarcoma-derived fibroblasts or bone stromal cells, in the presence of M-CSF, evidence of osteoclast formation was noted with formation of TRAP^+^ and VNR^+^ MNCs. In addition, co-cultures of either Ewing's sarcoma-derived fibroblasts or bone stromal cells and PBMCs on dentine slices resulted in the formation of lacunar resorption pits (Figure 2A–C). Resorption pit formation was abolished by the addition of OPG. In transwells cultures of Ewing's sarcoma-derived fibroblasts or bone stromal cells with PBMCs, no TRAP^+^ or VNR^+^ MNCs were formed and no lacunar resorption was noted on dentine slices.

### Expression of RANKL, OPG and TRAIL mRNA

**Figure 3:**
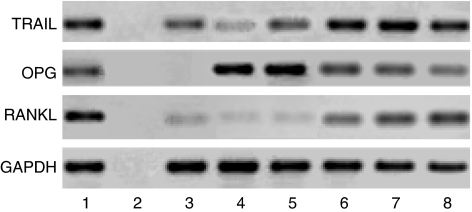
Expression of RANKL, OPG and TRAIL mRNA by fibroblasts derived from Ewing's sarcoma and TC71 cells. Reverse transcription-polymerase chain reaction products were fractionated on agarose gel. Lane 1, positive control; lane 2, negative control; lane 3, TC71; lanes 4–5, Ewing's sarcoma-derived fibroblasts from two patients; lanes 6–8, normal bone stromal cells.

Expression of RANKL, OPG and TRAIL was noted in both Ewing's sarcoma-derived fibroblasts and bone stromal cells (Figure 3). Significantly less expression of RANKL was noted in Ewing's sarcoma-derived fibroblasts than bone stromal cells. Reverse transcriptase–polymerase chain reaction studies also showed that Ewing's sarcoma tumour cells exhibited weak expression of the m-RNA for RANKL, OPG and TRAIL.

### Effect of TC71 Ewing's sarcoma cells on osteoclast formation

**Figure 4:**
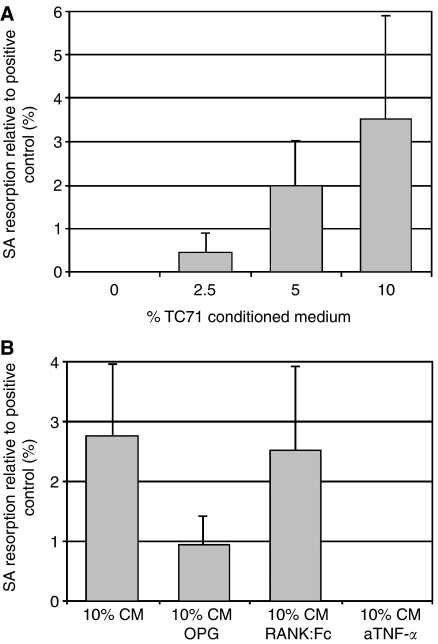
(**A**) % surface area (SA) resorption formed in human PBMC cultures incubated with M-CSF and TC71 conditioned medium relative to positive control (PBMC cultures with M-CSF and RANKL). Error bars denote s.e.m. (*n*=3). (**B**)The effect of OPG, RANK: Fc and neutralising antibodies to TNF-*α* on resorption in PBMC cultures incubated with M-CSF and 10% TC71 conditioned medium. The data represent the mean % surface area (SA) lacunar resorption relative to the positive control (PBMC cultures with M-CSF and RANKL). Error bars denote s.e.m. (*n*=6).

Cultures of human PBMCs incubated with M-CSF and TC71 CM resulted in the formation of TRAP^+^ and VNR^+^ MNCs capable of lacunar resorption. The amount of lacunar resorption was dose-dependent, with maximal resorption seen at CM concentration of 10% (Figure 4A). The addition of OPG or RANK: Fc to TC71 CM-treated PBMC cultures did not abolish osteoclast formation or significantly inhibit lacunar resorption. The addition of neutralising antibodies to TNF-*α* abolished lacunar resorption in TC71 CM-treated PBMC cultures (Figure 4B). Cultures of TC71 cells alone, both in the presence and absence of M-CSF/RANKL or M-CSF/TNF-*α*, did not result in the formation of TRAP^+^ or VNR^+^ cells in cultures on glass coverslips or in lacunar resorption in cultures on dentine slices.

## DISCUSSION

This study has shown that TAMs isolated from Ewing's sarcoma are capable of differentiation into mature functional osteoclasts. Mononuclear osteoclast precursors were found in the CD14^+^ (macrophage) population of cells isolated from Ewing's sarcomas and these cells were capable of differentiating into osteoclasts capable of lacunar resorption in the presence of RANKL and M-CSF. Both bone stromal cells and Ewing's sarcoma-derived fibroblasts expressed RANKL and were capable of inducing osteoclast formation by a contact-dependent mechanism. In the presence of M-CSF, TAM-osteoclast differentiation was also induced by TNF-*α*. Osteoclast formation was also induced by a soluble factor produced by TC71 Ewing's sarcoma cells. When Ewing's sarcoma cells were cultured directly on bone (both in the presence and absence of M-CSF and RANKL or TNF-*α*), these cells did not differentiate into osteoclasts and were not capable of lacunar resorption.

Our findings are in keeping with the concept that tumour cells are not capable of lacunar resorption and that osteoclasts (which are part of the mononuclear phagocyte system) are required for tumour osteolysis (Quinn *et al*, 1994; Clohisy *et al*, 1996; Clohisy and Ramnaraine, 1998). Osteoclast formation was not seen in cultures of tumour cells and was only noted in cultures that contained Ewing's sarcoma-derived TAMs or their monocyte precursors (McBride, 1986; Mantovani *et al*, 1992). Osteoclast formation is known to occur from the monocyte fraction of peripheral blood mononuclear cells as well as from tissue macrophages isolated from neoplastic and non-neoplastic lesions (Fujikawa *et al*, 1996; Quinn *et al*, 1998; Lau *et al*, 2006). Heterogeneity of monocytes and macrophages is well-recognised and these cells are capable of differentiation into specialised cells of the mononuclear phagocyte system (MPS), such as the osteoclast, the specialised MPS cell of bone (Gordon and Taylor, 2005). Osteoclasts are known to have a highly specific cytochemical and antigenic phenotype and are uniquely capable of lacunar bone resorption (Athanasou, 1996). We found that TAMs isolated from Ewing's sarcoma did not express the osteoclast markers TRAP and VNR, but did express CD14, a monocyte/macrophage marker not expressed by osteoclasts. When TAMs were incubated with M-CSF and RANKL, there was formation of numerous TRAP^+^/VNR^+^ MNCs that were capable of extensive lacunar resorption.

We found that Ewing's sarcoma-derived fibroblasts and bone stromal cells expressed RANKL and that these cells were capable of inducing osteoclast formation (in the absence of soluble RANKL) when cocultured in contact with monocyte-derived macrophages. This process was inhibited by the addition of OPG, a decoy receptor for RANKL. These findings indicate that this contact-dependent process is RANKL-driven, which means that osteoclast formation and osteolysis occurring in Ewing's sarcoma is through a cell–cell interaction involving RANK-expressing mononuclear phagocyte osteoclast precursors and RANKL-expressing tumour fibroblasts or bone stromal cells in the affected bone. Although the low level of RANKL expression by Ewing's sarcoma-derived fibroblasts raises the question of how significant is the contribution of this RANKL-mediated mechanism, fibroblasts and bone stromal cells have been shown to play a similar role in inducing osteoclast formation in other bone tumours, such as giant cell tumour of bone and Paget's sarcoma, as well as in tumours known to metastasise to bone, such as melanoma and neuroblastoma (Michigami *et al*, 2001; Lau *et al*, 2005; Lau *et al*, 2006; Sun *et al*, 2006). Tumour fibroblasts, like bone stromal cells, have also been shown to produce M-CSF, a survival factor for macrophages and an essential cofactor in osteoclastogenesis (Felix *et al*, 1994; Flanagan and Lader, 1998).

TNF-*α* is known to play a role in cell proliferation, and serum levels of TNF-*α* as well as M-CSF have been correlated with the progression of Ewing's sarcoma (Kwon *et al*, 1998; Holzer *et al*, 2003). Tumour necrosis factor-*α* has been shown to play a role in inducing osteoclast differentiation from marrow-derived circulating monocyte precursors and inflammatory macrophages (Kudo *et al*, 2002; Sabokbar *et al*, 2003), and we found that the addition of TNF-*α* (with M-CSF) to cultures of Ewing's sarcoma-derived TAMs induced osteoclast formation in the absence of RANKL. As Ewing's sarcoma cells are known to produce abundant TNF-*α* (Rube *et al*, 2003), we further investigated whether this cytokine played a role in stimulating osteoclast formation by tumour cells. We found that, in the presence of M-CSF and the conditioned medium of cultured TC71 Ewing's sarcoma cells, monocytes differentiated into TRAP^+^/VNR^+^ MNCs that were capable of lacunar resorption. This osteoclast formation was not inhibited by the addition of OPG but was abolished by an antibody to TNF-*α*. The MNCs that formed in these monocyte cultures were small and they produced discrete lacunar resorption pits that were morphologically similar to those formed in TNF-*α*-treated TAM cultures. As TNF-*α* is known to stimulate RANKL expression and as TC71 Ewing's sarcoma cells expressed RANKL, it was not possible to determine if TNF-*α* in the TC71 conditioned medium was directly inducing osteoclastogenesis. It is possible that the inhibitory effect of the TNF-*α* antibody on osteoclast formation may have been directed against the known permissive effect of this cytokine on RANKL-induced osteoclastogenesis (Lam *et al*, 2000). Although the soluble factor produced by TC71 cells did not induce as much osteoclast formation and resorption as RANKL, it could nevertheless play a significant role in the osteolysis of a highly cellular tumour such as Ewing's sarcoma where there would be numerous proliferating tumour cells producing this osteoclastogenic factor.

All the cellular and humoral elements which we have identified as playing a role in TAM-osteoclast differentiation can be found in the microenvironment of an osteolytic Ewing's sarcoma of bone. It is possible that the extent of TAM-osteoclast differentiation (and resultant osteolysis) that occurs in a Ewing's sarcoma is influenced by cytokines/growth factors produced by the various cellular components, which we have shown to influence this process. In keeping with this hypothesis, we found that OPG, which acts as a decoy receptor for RANK, inhibited RANKL-induced osteoclast formation and that an antibody to TNF-*α* inhibited osteoclast formation associated with the production of a soluble factor by Ewing's sarcoma cells. We also noted that the bisphosphonate, zoledronate, abolished osteoclast formation and resorption by osteoclasts produced from TAMs. Bisphosphonates are known to inhibit osteoclast formation and resorption activity and to induce osteoclast apoptosis (Rogers *et al*, 2000); bisphosphonates have also been shown to inhibit the growth of Ewing's sarcomas through mechanisms that involve upregulation of osteoprotegerin (Zhou *et al*, 2005). Bisphosphonates and other agents that influence osteoclast formation and resorption activity may thus be useful in controlling the tumour osteolysis associated with Ewing's sarcoma.

## Figures and Tables

**Table 1:** 

	**Primer sequence**	**Size of product (base pairs)**	**Annealing temperature (°C)**
GAPDH	Forward: 5′-CACTGACACGTTGGCAGTGG-3′;	360	60
	Reverse: 5′-CATGGAGAAGGCTGGGGCTC-3′;		
OPG	Forward 5′-ATGAACAAGTTGCTGTGCTG-3′;	354	58
	Reverse 5′-GCAGAACTCTATCTCAAGGTA-3′;		
RANKL	Forward 5′-CAGATGGATCCTAATAGAAT-3′;	324	56
	Reverse 5′-ATGGGAACCAGATGGGATGTC-3′;		
TRAIL	Forward 5′-ATCATGGCTATGATGGAGGT-3′;	315	58
	Reverse 5′-AACTGTAGAAATGGTTTCCTC-3′		

GAPDH=glyceraldehyde-3-phosphate dehydrogenase; OPG=osteoprotegerin; RANKL=receptor activator for nuclear factor-κB ligand; TRAIL=tumour necrosis factor related apoptosis inducing ligand.
